# miR-1 Exacerbates Cardiac Ischemia-Reperfusion Injury in Mouse Models

**DOI:** 10.1371/journal.pone.0050515

**Published:** 2012-11-30

**Authors:** Zhenwei Pan, Xuelin Sun, Jinshuai Ren, Xin Li, Xu Gao, Chunying Lu, Yang Zhang, Hui Sun, Ying Wang, Huimin Wang, Jinghao Wang, Liangjun Xie, Yanjie Lu, Baofeng Yang

**Affiliations:** 1 Department of Pharmacology (Key Laboratory of Cardiovascular Medicine Research, Ministry of Education; State-Province Key Laboratories of Biomedicine-Pharmaceutics of China), Harbin Medical University, Harbin, Heilongjiang, People’s Republic of China; 2 Departments of Biochemistry, Harbin Medical University, Harbin, Heilongjiang, People’s Republic of China; Virginia Commonwealth University Medical center, United States of America

## Abstract

Recent studies have revealed the critical role of microRNAs (miRNAs) in regulating cardiac injury. Among them, the cardiac enriched microRNA-1(miR-1) has been extensively investigated and proven to be detrimental to cardiac myocytes. However, solid *in vivo* evidence for the role of miR-1 in cardiac injury is still missing and the potential therapeutic advantages of systemic knockdown of miR-1 expression remained unexplored. In this study, miR-1 transgenic (miR-1 Tg) mice and locked nucleic acid modified oligonucleotide against miR-1 (LNA-antimiR-1) were used to explore the effects of miR-1 on cardiac ischemia/reperfusion injury (30 min ischemia followed by 24 h reperfusion). The cardiac miR-1 level was significantly increased in miR-1 Tg mice, and suppressed in LNA-antimiR-1 treated mice. When subjected to ischemia/reperfusion injury, miR-1 overexpression exacerbated cardiac injury, manifested by increased LDH, CK levels, caspase-3 expression, apoptosis and cardiac infarct area. On the contrary, LNA-antimiR-1 treatment significantly attenuated cardiac ischemia/reperfusion injury. The expression of PKCε and HSP60 was significantly repressed by miR-1 and enhanced by miR-1 knockdown, which may be a molecular mechanism for the role miR-1 in cardiac injury. Moreover, luciferase assay confirmed the direct regulation of miR-1 on protein kinase C epsilon (PKCε) and heat shock protein 60 (HSP60). In summary, this study demonstrated that miR-1 is a causal factor for cardiac injury and systemic LNA-antimiR-1 therapy is effective in ameliorating the problem.

## Introduction

MicroRNAs (miRNAs) are a group of single strand non-coding RNAs that inhibit the translation of protein-coding genes by annealing inexactly to complementary sequences in the 3′UTRs of target mRNAs [1]. Recent studies indicated that miRNAs are broadly involved in the development of cardiovascular diseases, including arrhythmia, hypertrophy, heart failure and cardiac injury etc [2]. The regulatory action of miRNAs is often physiologically significant that modulation of expression of a single miRNA could change a specific pathological process [2]. Several miRNAs have been proven to be importantly involved in the pathogenesis of cardiac ischemia-reperfusion injury, and interfering their expression is able to alleviate cardiac injury, underscoring the potential of miRNAs as anti-ischemic targets [3], [4].

The muscle-specific miRNA miR-1 is one of the miRNAs shown to play a role in cardiac injury [5], [6], [7]. miR-1 is the first miRNA that has been extensively explored and confirmed to be a key regulator of cardiac development and disease [7], [8], [9], [10], [11]. In an earlier study, Zhao *et al* found that miR-1 participates in cardiogenesis by regulating the expression of a transcription factor Hand2 [8]. Our group discovered that miR-1 promotes cardiac ischemic arrhythmias by targeting KCNJ2 gene, which encodes Kir2.1 inward rectifier K^+^ channel protein subunit, and GJA1 gene encoding connexin-43 gap junction channel protein subunit [9]. Sayed *et al* demonstrated that miR-1 inhibits cardiac hypertrophy by affecting the growth-related targets, including Ras GTPase-activating protein (RasGAP), cyclin-dependent kinase 9 (Cdk9), fibronectin, and Ras homolog enriched in brain (Rheb) [10]. After that, several studies showed that miR-1 exacerbates cardiac injury by affecting the expression of a host of protective proteins, e.g. BCL2, HSP60, insulin growth factor 1(IGF-1), etc [6], [7], [11]. However, these previous studies dealt with transient alterations of miR-1 expression and the effects of long-term overexpression of miR-1 on cardiac injuries have not been studied. In addition, accumulating evidence has highlighted the potential of miRNA knockdown approach in preventing cardiac injury [12], [13]. In this study we employed both gain- and loss-of-function approaches to elucidate the roles of miR-1 in cardiac injuries and the therapeutic potential of miR-1 knockdown. To this end, we generated a cardiac-specific miR-1 over-expression mouse line and employed the LNA-antimiR-1-mediated miR-1 knockdown technique.

## Materials and Methods

### Ethics Statement

All experimental procedures were in accordance to the Institutional Animal Care and Use Committee of Harbin Medical University, P.R. China. The protocol was approved by the Experimental Animal Ethic Committee of Harbin Medical University, China (Animal Experimental Ethical Inspection Protocol No. 2010102). The surgery procedures were performed under sodium pentobarbital anesthesia.

**Figure 1:**
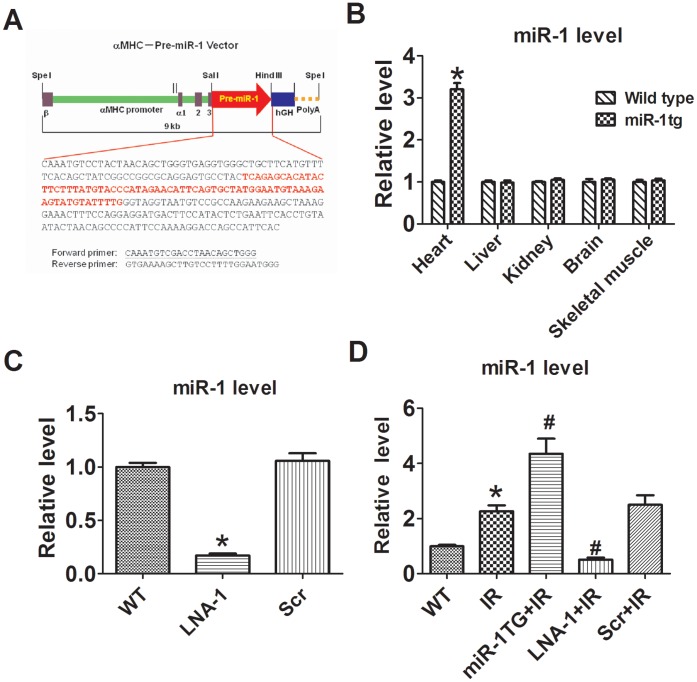
Generation of miR-1 transgenic mice and the detection of miR-1 levels. Schematic illustration of generation of miR-1 transgenic mice (A), level of miR-1 in miR-1 transgenic mice (B), LNA-1 treated mice (C), and ischemia reperfusion (IR) hearts of mice (D). Src, scramble locked nucleic acid. Data are expressed as mean±SEM; n = 5; *P<0.05 *vs* wild type (WT); # P<0.05 *vs* IR.

**Table 1:** Echocardiography of wild type (WT) and miR-1 transgenic (miR-1 Tg) mice.

Group	LVDd(mm)	LVSd(mm)	IVSd(mm)	IVSs(mm)	FS (%)	EF (%)
WT	3.18±0.37	1.92±0.29	0.98±0.17	1.33±0.24	39.6±4.4	76.6±5.5
miR-1 tg	3.48±0.88	2.17±0.89	0.9±0.2	1.2±0.18	40.2±14.4	74.6±14.3

EF, eject fraction; FS, fractional shortening; LVDd, left ventricle diastolic diameter; LVSd, left ventricle systolic diameter; IVSd, interventricular septum diastolic thickness; IVSs interventricular septum systolic thickness. Data are expressed as mean±SD; n = 6 for each group.

### Animals

Adult male C57BL/6 mice (22–25 g) were used in this study. Mice were kept under standard animal room conditions (temperature 21±1°C; humidity 55–60%) with food and water *ad libitum* for one week before the experimental procedures.

### Generation of miR-1 Transgenic Mice

A fragment of DNA containing the precursor sequence of mmu-miR-1a-2 was amplified and subcloned into the Sal I and Hind III sites of the Bluescript vector (Promega) carrying the cardiac-specific α myosin heavy chain (αMHC) promoter and human growth hormone poly(A) signal. The pre-miR-1 sequence flanked by 5′end αMHC promoter and 3′end poly(A) was obtained by digestion and extraction, which was then injected into fertilized eggs of C57BL/6 mice. The injected eggs in a group of 10–15 were implanted bilaterally into the oviduct of the pseudo-pregnant females. The positive miR-1 transgenic mice were identified by the successful PCR amplification of αMHC. The forward primer is 5′-CCTTACCCCACATAGACCT-3′ and the reverse primer is 5′-CTTAGCAGGTCCATATGGGC-3′.

### Echocardiographic Measurement

Transthoracic echocardiography with an ultrasound instrument (Vivid 7 GE Medical) equipped with a 10-MHz phased-array transducer was used to measure the left ventricular function. Left ventricular systolic diameter (LVSd), left ventricular diastolic diameter (LVDd), interventricular septum diastolic thickness (IVSd) and interventricular septum systolic thickness (IVSs) were measured, and left ventricular ejection fraction (LVEF) and fractional shortening (FS) were calculated from M-mode recording.

**Figure 2:**
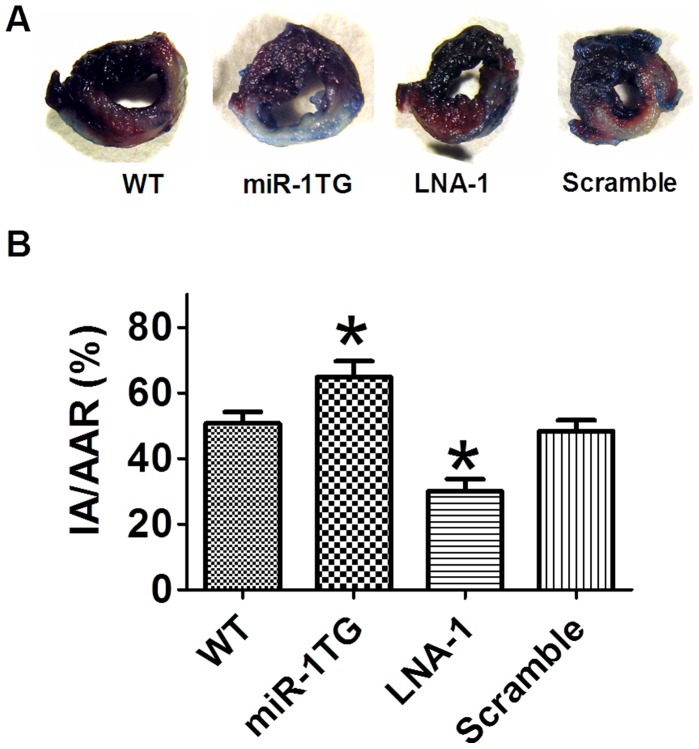
Effects of miR-1 on cardiac infarct area of mice after ischemia/reperfusion injury in mice. A. Representative images showing infarct areas; B. Statistical analysis of IA/AAR ratio. LNA-1, LNA-antimiR-1; Scramble, scramble locked nucleic acid; IA, infarct area; AAR, area at risk. Data are expressed as mean±SEM; n = 8; *P<0.05 *vs* WT.

### Cardiac Ischemia-reperfusion Injury

Adult male C57BL/6 mice were orally intubated with 20-gauge tube and ventilated (mouse ventilator, UGO BASILE, Biological Research Apparatus, Italy) at a respiratory rate of 100 breaths/min and a tidal volume of 0.3 ml. A left-sided thoracotomy was performed and the heart was exposed. A 7-0 silk thread was then passed through the left anterior descending coronary artery (LAD) near its origin. Both ends of the thread were passed through a plastic cannula to allow ligation and reopening of the coronary artery. Sham-operated animals underwent an identical procedure except that the thread was passed through the myocardium without tying. Myocardial ischemia-reperfusion (I/R) was accomplished by 30 min ischemia and 24 h reperfusion. At the end of the myocardial I/R protocols, the LAD was briefly re-occluded and 0.3 ml Evans blue dye (5%, Sigma Aldrich) was injected retrogradely into the vena cava to delineate the region of myocardial perfusion. After washing out remaining blood and trimming the right ventricle, the left ventricle was cut into 2 mm thick slices and stained with 1% triphenyltetrazolium chloride (TTC) for 10 min at 37°C, and the infarct area was stainless while the live area turned red. The area at risk (ischemic area) and infarct area was calculated using Image ProPlus 5.0 software. For further study, the tissues in ischemic area of the hearts were collected and stored at −80°C.

### Synthesis and Administration of Locked Nucleic Acid Antisense miR-1

The antisense sequence of miR-1 (LNA-antimiR-1) was synthesized by Exiqon (Denmark) and five nucleotides or deoxynucleotides at both ends of the antisense molecules were locked (LNA; the ribose ring is constrained by a methylene bridge between the 2′-*O*- and the 4′-*C* atoms). The sequence of LNA-antimiR-1 is 5′-ACTTCTTTACATTCC-3′. A scrambled sequence was used as negative control: 5′-ACGTCTATACGCCCA-3′. LNA-antimiR-1 or negative control sequence at a dose of 1 mg/kg was intravenously injected via tail vein into mice three days before cardiac I/R.

**Figure 3:**
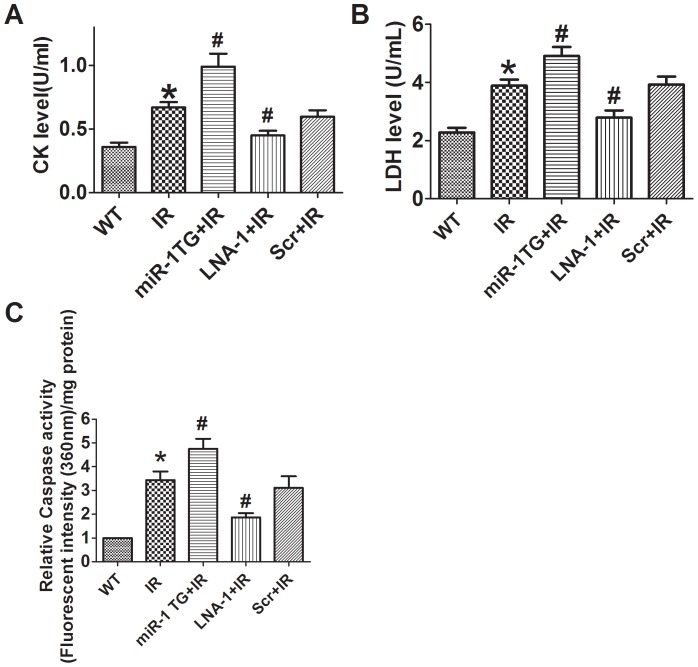
Effects of miR-1 on serum creatinine kinase, lactate dehydrogenase level and cardiac caspase-3 activity after IR injury in mice. A, serum creatine kinase; B, lactate dehydrogenase; C, caspase-3 activity. Data are expressed as mean±SEM; n = 8 for LDH and CK, n = 5 for caspase-3; *P<0.05 *vs* WT, #P<0.05 *v*s IR.

**Figure 4:**
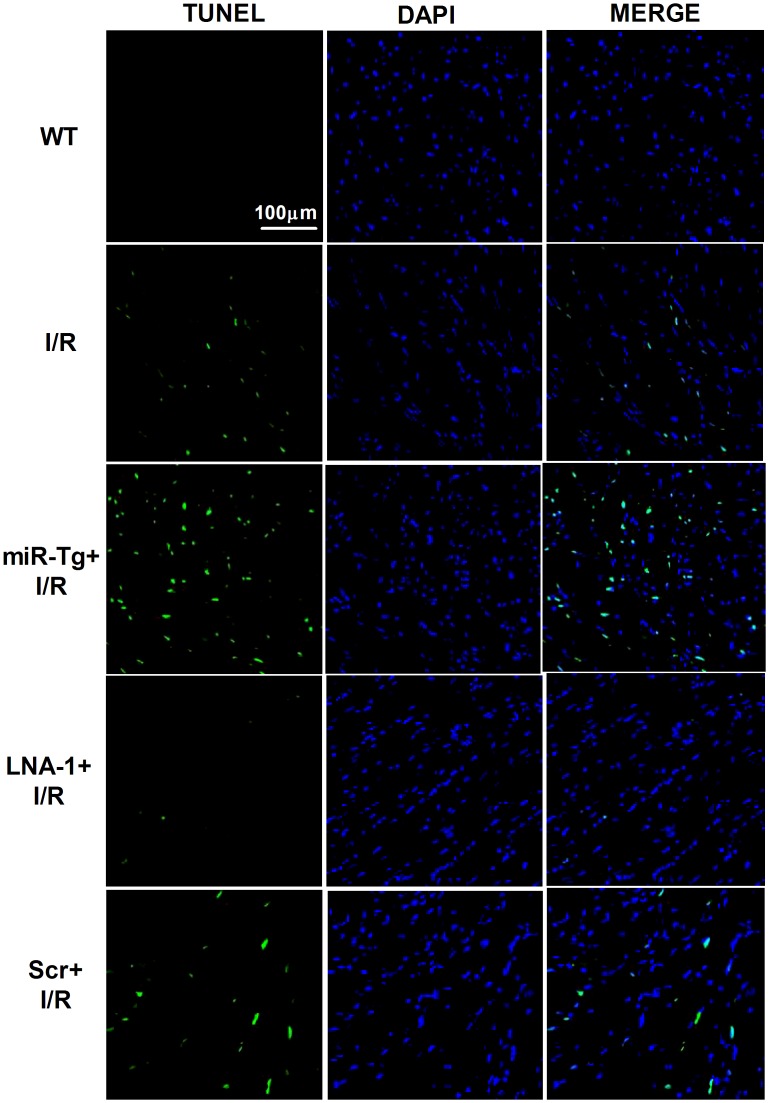
Effects of miR-1 and LNA-1 on cardiomyocyte apoptosis in heart subjected to ischemia/reperfusion (I/R) injury by TUNEL staining. WT, wild type; miR-1 Tg, miR-1 transgenic; LNA-1, LNA-antimiR-1; Scr, Scrambled LNA sequence. Apoptotic cells are stained in green.

### Western Blot

Total protein was extracted from the left ventricle of mice for immunoblotting analysis with the procedures described in detail elsewhere [9]. Briefly, protein samples (80 µg) were separated in SDS-PAGE and blotted to nitrocellulose membrane. The blots were probed with primary antibody including PKCε (1∶200 dilution, Santa Cruz, USA), HSP60 (1∶200 dilution, Santa Cruz, USA) and β-actin (1∶200 dilution, Santa Cruz, USA), and then secondary antibody (Alexa Fluor). The western blot bands were collected by Imaging System (LI-COR Biosciences, Lincoln, NE, USA) and quantified with odyssey v1.2 software by measuring intensity (area×OD) in each group with β-actin as an internal control.

### qRT-PCR

Total RNA from cardiac tissues were extracted using Trizol reagent (Invitrogen,USA) according to manufacturer’s protocols. The level of miR-1 was measured using TaqMan MicroRNA Assay Kit (Applied Biosystems, Foster City, CA, USA) with U6 as an internal control.

**Figure 5:**
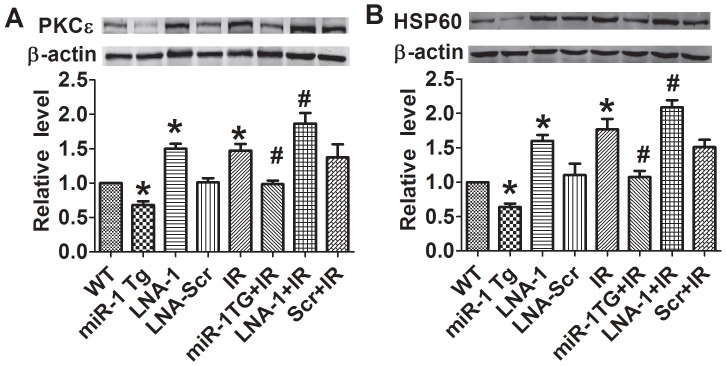
Expression of PKCε and HSP60 in miR-1Tg and LNA-antimiR-1 (LNA-1) treated mice before and after ischemia/reperfusion injury. A, PKCε, protein kinase C epsilon; B, HSP60, heat shock protein 60. Data are expressed as mean±SEM; n = 5; *P<0.05 *v*s WT, #P<0.05 *vs* IR.

### Serum CK and LDH Assay

Serum CK and LDH were measured using CK and LDH detection kits (Nanjing Jiancheng Bioengineering Institute) according to the manufacturer’s instruction.

### Caspase-3 Activity Assay

Caspase-3 activity was measured using CaspACE™ Assay System (fluorometric kit, Promega, USA) according to the manufacturer’s instruction.

**Figure 6:**
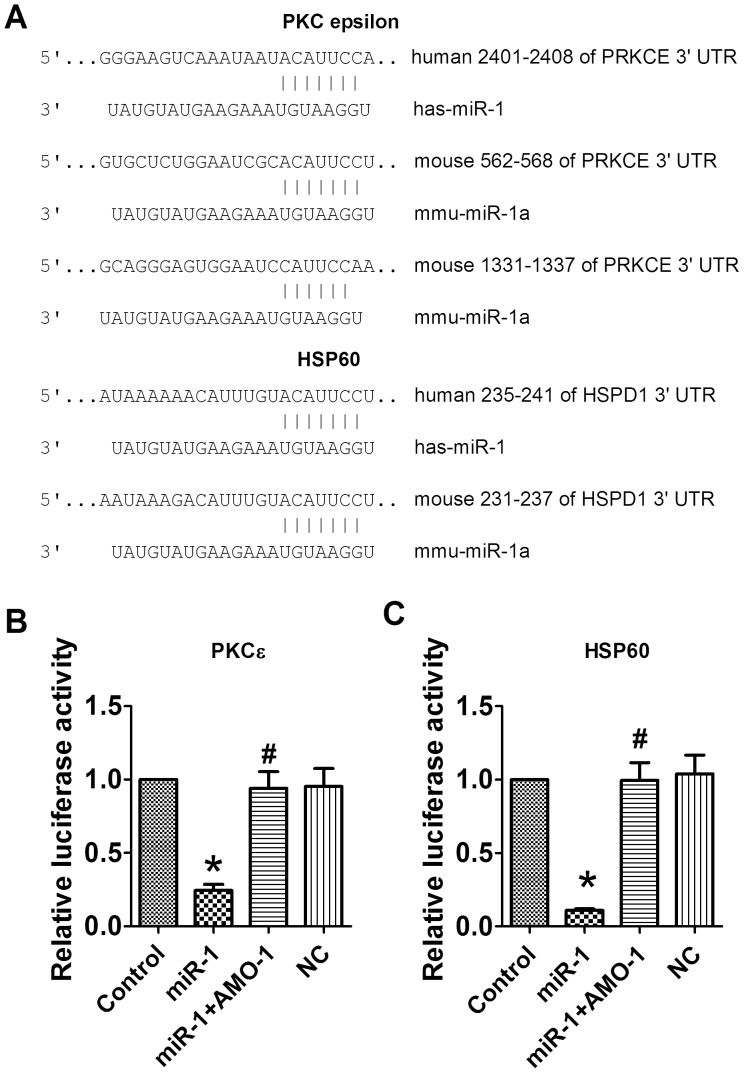
Verification of PKCε and HSP60 as targets of miR-1. A. Sequence alignment between miR-1 and the 3′UTRs of PKCε and HSP60 of human and mouse. B, C. Luciferase reporter activities of chimeric vectors carrying luciferase gene and a fragment of PKCε or HSP60 3′UTR containing the binding sites of miR-1. Data are expressed as mean±SEM; n = 4; *P<0.05 *vs* control, #P<0.05 *vs* miR-1.

### TUNEL Staining

Apoptosis of cardiac myocytes was detected by staining mouse heart cryosections with the In situ Cell Death Detection Kit (TUNEL fluorescence FITC kit, Roche, Indianapolis, IN, USA) according to the manufacturer’s instruction. After TUNEL staining, the heart sections were immerged into DAPI (Sigma-Aldrich) solution to stain nuclei. Fluorescence staining was viewed by a laser scanning confocal microscope (FV300, Olympus, Japan).

### Synthesis of miR-1 and anti-miR-1 Antisense Inhibitor (AMO-1)

miR-1 and its antisense oligonucleotidesAMO-1 were synthesized by GenePharma (Shanghai GenePharma Co., Ltd). Additionally, a scrambled RNA was used as a negative control (NC); miR-1, sense: 5′-UGGAAUGUAAAGAAGUGUGUAU-3′ and antisense: 5′-AUACACACUUCUUUACAUUCCA-3′. All pyrimidine nucleotides in the NC or miR-1 were substituted by their 2′-*O*-methyl analogues to improve RNA stability.

### Luciferase Assay

To construct reporter vectors bearing miRNA-target sites, we first obtained fragments of the 3′UTRs of HSP60, 70 and PKCε containing the exact target sites for miR-1 by PCR amplification. 3′UTR fragments were inserted into the multiple cloning sites downstream the luciferase gene (HindIII and SacI sites) in the pMIR-REPORTTM luciferase miRNA expression reporter vector (Ambion, Inc.) to form chimeric plasmid.

After that, 1 µg of the chimeric plasmid (firefly luciferase vector), 0.1 µg PRL-TK (TK-driven Renilla luciferase expression vector) and the appropriate miRNAs or their inhibitors were co-transfected with lipofectamine 2000 (Invitrogen) into HEK-293 cells (1×10^5^/well). Luciferase activities were measured with a dual luciferase reporter assay kit (Promega) on a luminometer (GloMax™ 20/20), 48 h following transfection. For all experiments, transfection took place 24 h after starvation of cells in serum-free medium. The normalized luciferase activity relative to control group was used to demonstrate the alteration of mRNA transcription.

### Statistical Analysis

All data were expressed as mean±SEM. Statistical analysis was performed using one-way ANOVA followed by Dunnett’s *t*-test. Differences were considered as statistically significant when P<0.05.

## Results

### Overexpression and Knockdown of miR-1 in Mice

In this study, a cardiac specific miR-1 transgenic mouse line was established by inserting the precursor sequence of mmu-miR-1a-2 incorporated with the cardiac-specific α myosin heavy chain (αMHC) promoter into the mouse genome (Fig. 1A). Cardiac specific transgenic overexpression of miR-1 in mice led to a 3.2 fold increase in cardiac miR-1 level with no changes in liver, kidney, brain and skeletal muscle, indicating the successful establishment of miR-1 Tg mice (Fig. 1B). Moreover, miR-1 overexpression in the heart from two months old miR-1 transgenic mice caused no functional and structural alterations as indicated by echocardiographic measurement (Table 1). LNA-antimiR-1 was used to achieve *in vivo* knockdown of endogenous miR-1. LNA-antimiR-1, administered via tail vein at a dose of 1 mg/kg, significantly reduced miR-1 expression in the heart by about 83% (Fig. 1C).

### Effects of Gain- and Loss-of-function of miR-1 on Infarct Size of I/R Hearts

As shown in Fig. 1D, miR-1 levels increased by 2.3 fold in wild type and 4.3 fold in miR-1 Tg mice when subjected to I/R, while LNA-1 treatment decreased miR-1 level in mice with I/R injury. To examine the role of miR-1 in cardiac injury, we measured infarct size of hearts with TTC and Evans blue staining. As shown in Fig. 2A, infarct size was larger in miR-1 Tg mice than in WT controls 24 h after reperfusion, while LNA-antimiR-1 treatment reduced infarct size. The area at risk (AAR) was nearly the same among groups (data not shown). The IA/AAR ratio was significantly increased from 50.6±3.6% (WT) to 64.9±4.8% (miR-1 Tg). LNA –antimiR-1 reduced the IA/AAR ratio to 30.1±3.7% (Fig. 2B). These results indicate that miR-1 produces harmful effects on the heart cardiac I/R, while knockdown of miR-1 produces protective effects against I/R injury.

### Effects of miR-1 on Creatinine Kinase (CK), Lactate Dehydrogenase (LDH) Release, Cardiac Caspase-3 Activity and Cardiomyocyte Apoptosis after IR Injury

Consistent with cardiac infarct size result, serum CK and LDH were robustly released after IR injury, which was further increased in miR-1 overexpression mice. On the contrary, knockdown of miR-1 by LNA-antimiR-1 inhibited the increases of serum CK and LDH levels caused by IR injury (Fig. 3A, B). Furthermore, we examined the effects of miR-1 on caspase-3 activity of ischemic heart. We found that miR-1 overexpression enhanced caspase-3 activity after IR injury. In contrast, LNA-antimiR-1 treatment alleviated the activation of caspase-3 (Fig. 3C). We further examined the apoptosis of cardiomyocytes subjected to I/R injury by TUNEL staining. Compared with wild type control, TUNEL positive cells were increased in miR-1 Tg mice. LNA-antimiR-1 markedly decreased apoptosis of cardiomyocytes, while scramble LNA produced no effects (Fig. 4).

### Expression of PKCε and HSP60 in miR-1 Tg and LNA-antimiR-1 Treated Mice before and after I/R Injury

To exploit the underlying mechanisms for the harmful effects of miR-1 on the heart, we evaluated the influence miR-1 on PKCε and HSP60 expression which are known to play a protective role against cardiac injury. In miR-1 Tg mice, the expression of cardiac PKCε was significantly inhibited (Fig. 5A), while in mice administered with LNA-antimiR-1 it was increased (Fig. 5A). When the mice were subjected to I/R, cardiac PKCε level was increased. However, in miR-1 Tg mice the increase was suppressed and in LNA-antimiR-1 treated mice PKCε level was elevated (Fig. 5A). In terms of HSP60, qualitatively the same alterations were observed (Fig. 5B). These results demonstrated that the detrimental action of miR-1 on the heart may be mediated by repressing the expression of PKCε and HSP60.

### Identification of PKCε and HSP60 as the Targets of miR-1

PKCε and HSP60 are bioinformatically predicted to be the conservative targets of miR-1 by Targetscan. The miRNA:mRNA complementary between miR-1 and the 3′UTRs of PKCε and HSP60 are shown in Fig. 6A. To experimentally establish these genes as target of miR-1, we subcloned the 3′UTRs of PKCε and HSP60 into the 3′UTR of a luciferase plasmid to construct chimeric vectors. Cotransfection of the chimeric vectors with miR-1 (Fig. 6B, C) resulted in lower luciferase activity relative to the transfection of chimeric vectors alone. The repression of PKCε and HSP60 transcription activities by miR-1 was alleviated by co-application of AMO-1. The negative control sequences produced no effects on luciferase activity of the chimeric vectors. These results indicate that PKCε and HSP60 are direct targets of miR-1.

## Discussion

Previously studies indicate that miR-1 may be a critical factor in cardiac injury. The expression of miR-1 was strongly increased in both serum, cardiac tissues and cultured cardiac myocytes during cardiac injury induced by various stimuli [6], [7], [9], [11]. In rats, serum miR-1 level is increased after myocardial infarction, which strongly correlates with infarct size and can be significantly reduced by ischemic preconditioning [14]. We and others found that serum level of miR-1 was closely related to cardiac injury in acute myocardial infarction (AMI) patients [14], [15]. Circulating levels of miR-1 are significantly increased in patients with AMI, which positively correlates with serum CK-MB level [14], a marker of ischemic myocardial damage. Further study showed that exogenous overexpression of miR-1 in cultured cardiac myocytes exacerbates H_2_O_2_-induced injury and knockdown of miR-1 expression produces protective effects [7]. Yu *et al* demonstrated that overexpression of miR-1 abrogates insulin growth factor 1 (IGF-1)-mediated protection against glucose-induced cardiomyocyte injury *in vitro* [6]. Consistent with previous *in vitro* reports, this study provided *in vivo* data supporting the detrimental role of miR-1 on cardiac I/R injury in mice. We successfully established cardiac-specific miR-1 overexpression mice, which are more sensitive to I/R stress than wild-type controls. In contrast, knockdown of miR-1 with LNA-antimiR-1 alleviated cardiac I/R injury. Interestingly, Zhao *et al* observed increased proliferation of cardiac myocytes at postnatal day 10 of in mice with miR-1-2 knockout [16], indicating that miR-1 is pro-survival molecule.

Generally, miRNAs exert their function by repressing the translation of proteins that are critical for pathophysiological processes, and one miRNA can act on multiple target genes [1]. Among the predicted targets of miR-1 identified by computational analysis, PKCε and HSP60 are highly conserved across species which are known to mediate cardioprotective effects. PKCε is a member of PKC family that plays a critical role in protecting the heart from I/R injury [17], [18], [19]. Upon activation, PKCε is translocated to the mitochondria where it phosphorylates mitochondrial proteins, e.g. aldehyde dehydrogenase cytochrome c oxidase subunit IV to confer cardioprotection [20], [21]. Heat shock protein 60 (HSP60) is a mitochondrial chaperon that is typically responsible for the transportation and refolding of proteins from the cytoplasm into the mitochondrial matrix and the replication and transmission of mitochondrial DNA [22], [23]. The expression of genes encoding HSP60 is upregulated upon heat shock response, which allows for the maintenance of other cellular processes occurring in the cell, especially during stressful conditions and thus protects the cell from damage [24]. Overexpression of HSP60 protects against apoptosis of cardiac myocytes, accompanied by decreases in mitochondrial cytochrome c release and caspase-3 activity and increases in activities of complex III and IV in mitochondria after ischemic stress [25], [26], indicating a significant role of HSP60 in mitochondrial function recovery and protection of stressed myocytes. In this study, we experimentally established PKCε and HSP60 as targets of miR-1 in mice, which is in line with the detrimental role of miR-1 in the heart. Consistently, the study reported by Shan *et al* demonstrated that miR-1 inhibits the expression of HSP60 in rat cardiomyocytes [5]. Moreover, in a previous study, our lab demonstrated that miR-1 overexpression induces adverse structural remodeling and impaired cardiac contractile function by targeting calmodulin (CaM) and cardiac myosin light chain kinase (cMLCK) in 4- and 6-month old miR-1 transgenic mice, but not 2-month old mice [27]. In the present study, 2-month old mice were used to avoid the influences of structural remodeling and function on I/R. Considering the multiple target property of a miRNA, it is speculated that other proteins related to cardiac injury may also be regulated by miR-1 directly or indirectly. In line with this view, the pro-survival BCL-2 [7] and IGF-1 [6] have been verified to be targets of miR-1. It is therefore conceivable that miR-1 produces its cardiac damaging effects by repressing expression of multiple survival target genes during cardiac injury. Further studies are required to obtain the full picture of the target network of miR-1 related to cardiac injury.

LNA-antimiR and cholesterol conjugated antagomir are two main kinds of therapeutic agents used to silence endogenous miRNA *in vivo*. The dose of antagomir required to achieve significant knockdown of miRNAs appears to be higher than that of LNA-antimiR. To successfully repress liver miR-122 expression in mice, an antagomir dose of 80 mg/kg was required [28], while LNA-antimiR at the dose of 6.25 mg/kg was able to produce equivalent effects [29]. In a recent study, LNA-antimiR-15b was shown to reduce cardiac miR-15b expression by 72% at a dose of as low as 0.5 mg/kg when given intravenously [12]. The high efficacy of LNA-antimiR indicates it a highly promising candidate for miRNA-based therapy. Interestingly, in this study we found that LNA-antimiR-1 inhibited miR-1 expression by 83% at a dose of 1 mg/kg and exerted apparent cardiac protective effects, reflecting the potential of LNA-antimiR-1 as a therapeutic agent against cardiac injury clinically.

In conclusion, this study demonstrated that miR-1 aggravated cardiac ischemia/reperfusion injury via inhibiting pro-survival proteins, e.g. PKCε and HSP60. Knockdown of miR-1 by LNA-antimiR-1 represents a new strategy in treating cardiac injury.
